# The Potential and Challenges of Focused Ultrasound-Mediated Therapies in the Management of Liver and Biliary Tract Cancers

**DOI:** 10.3390/cancers18101654

**Published:** 2026-05-20

**Authors:** Mira Florea, Viorica Nagy, Paul Milan Kubelac, Adrian Bartos, Delia Dima, Rares Potcoava Buiga, Monica Lupsor-Platon

**Affiliations:** 1 Institute of Advanced Studies in Science and Technology, Babeș-Bolyai University, 400347 Cluj-Napoca, Romania; lia.florea@ubbcluj.ro (M.F.); viorica.nagy@ubbcluj.ro (V.N.); delia.dima@ubbcluj.ro (D.D.); rares.buiga@ubbcluj.ro (R.P.B.); 2 Faculty of Medical and Health Sciences, Babeș-Bolyai University, 400347 Cluj-Napoca, Romania; 3 Oncologic Institute “Prof. Dr. Ion Chiricuță”, 400015 Cluj-Napoca, Romania; 4 Medical Imaging Department, “Iuliu Hatieganu” University of Medicine and Pharmacy, 400012 Cluj-Napoca, Romania; monica.lupsor@umfcluj.ro; 5 Medical Imaging Department, Regional Institute of Gastroenterology and Hepatology, 400162 Cluj-Napoca, Romania

**Keywords:** high intensity focused ultrasound (HIFU), histotripsy, low intensity focused ultrasound (LIFU), sono-chemotherapy, sonodynamic therapy (SDT), radiosensitization, sono-immunotherapy, abscopal effect, sonobiopsy, hepatobiliary cancers

## Abstract

Focused ultrasound (FUS)-mediated therapies have evolved with the advent of modern ultrasound-guided technology and MRI imaging, moving from their initial use as thermal ablation to a multifunctional platform for thermal and non-thermal ablation, immunomodulation, and targeted drug delivery. This narrative review explores the potential, limitations, and challenges of ablative high-intensity focused ultrasound (HIFU) therapies: HIFU thermal ablation and non-thermal ablation, histotripsy, as well as non-ablative low-intensity focused ultrasound (LIFU) applications in the management of hepatobiliary cancers. HIFU and histotripsy are reviewed as alternative or complementary treatment options in liver tumors, as well as their potential as bridging therapy. Histotripsy is addressed as a theranostic tool, not only by combining ablation with real-time ultrasound imaging guidance, but also by integrating it with sonobiopsy. It facilitates a liquid sonobiopsy of the ablated tumor by releasing intact tumor antigens and damage-associated molecular patterns, leading to potential molecular profiling. LIFU-induced targeted drug delivery (sono-chemotherapy), sonodynamic therapy, radiosensitization, immunomodulation of the immunosuppressive tumor microenvironment (sono-immunotherapy), and the potential to enhance the effect of immune checkpoint inhibitors in these malignancies are discussed. Since FUS-assisted procedures exhibit dual actions through therapeutic functionality associated with intra- and post-procedural ultrasound imaging guidance, they could have value as a theranostic tool in hepatobiliary interventional oncology. Although promising, the available clinical evidence for FUS-mediated therapies in hepatobiliary malignancies consists predominantly of early-stage feasibility studies, retrospective observational cohorts, and non-randomized comparative analyses. Further studies focused on standardized protocols, validation through large-scale, multicenter, prospective randomized clinical trials comparing FUS-based therapies with established treatments, and long-term follow-up of oncological efficacy could define their future role in multimodal oncological strategies.

## Simple Summary

There is growing interest in developing new therapeutic approaches for hepatobiliary cancers recognized as resistant to conventional therapies and prone to recurrence, despite current oncological advances. This paper reviews the potential, limitations and challenges of focused ultrasound (FUS)-mediated ablative therapies: high-intensity focused ultrasound (HIFU) thermal ablation and non-thermal ablation, histotripsy, as well as non-ablative low-intensity focused ultrasound (LIFU) modalities in the management of these cancers. The synergistic effect of combining acoustically mediated therapies with standard treatments, as part of multimodal oncological strategies, is also analyzed. Although it offers noninvasive, incision-free, radiation-free, and precise, image-guided treatment options, there are still hindrances to translating FUS-assisted therapies into clinical practice, such as technical limitations in targeting deep, difficult-to-access tumors, high treatment costs, limited availability of these technologies, and the need for large-scale evidence.

## 1. Introduction

Liver cancer contributes significantly to global cancer-related mortality, being the third leading cause of cancer-related deaths, with a 5-year survival rate of only 18% [1,2]. Primary liver cancer includes hepatocellular carcinoma (HCC), intrahepatic cholangiocarcinoma, hepatic angiosarcoma (a rare blood vessel cancer), and hepatoblastoma (a rare cancer in children). HCC is the most common (85%) and the sixth most frequently diagnosed cancer [2]. It arises from hepatocytes, often being linked to chronic liver diseases. Extrahepatic cholangiocarcinoma and gallbladder cancer are biliary tract tumors (BTC) developed in the epithelium of the bile ducts, accounting for 10% to 20% of cases. There is a hybrid form, a combined hepatocellular-cholangiocarcinoma tumor containing both hepatocellular and cholangiocellular components [3]. Due to the reduced symptomatology and suboptimal surveillance of chronic viral hepatitis B and C, alcohol-associated liver disease (ALD) and metabolic dysfunction-associated steatotic liver disease (MASLD) at risk of developing HCC, 50–60% of patients will be diagnosed at, or progress to, an advanced stage [4,5,6]. Curative therapies (resection, ablation, transplantation) are applicable in 25% of HCC patients, with >60-month median survival in early stages [7].

Cholangiocarcinoma (CCA) is a dense fibrotic neoplasia frequently diagnosed in the advanced stage as malignant biliary obstruction (MBO) with poor prognosis and limited curative options. Gallbladder adenocarcinoma is the fifth most common malignancy of the gastrointestinal tract and is found incidentally in 1–3% of cholecystectomy specimens [8]. Liver metastases (LMs) are more common than HCC [9], accounting for 64% of advanced liver cancer. More than half (53.1%) of LMs originate from the colorectum, followed by pancreatic and breast, lung cancer, neuroendocrine tumors and melanoma [10]. Hepatobiliary malignancies are heterogeneous solid tumors characterized as “cold tumors” with low immunogenicity and an immunosuppressive tumor microenvironment (TME) that limits the efficacy of systemic therapies. The treatment strategies of liver and biliary tract tumors include a combination of local therapies, with systemic chemotherapy, immunotherapy and molecularly targeted drugs [11,12,13,14].

The conventional local therapies include surgical resection, radiofrequency ablation (RFA), microwave ablation (MWA), transarterial chemoembolization (TACE), transarterial radiological embolization (TARE), radiotherapy, selective internal radiation therapy, stereotactic body radiation therapy (SBRT), and liver transplantation.

Systemic treatments in hepatobiliary malignancies have made increasing progress in terms of effectiveness in reducing tumor growth and the risk of metastases. However, tumor heterogeneity and multidrug resistance result in a recurrence rate of up to 70% after initial treatment. Moreover, conventional chemotherapy drugs induce severe systemic toxicity because of their nonspecific distribution, further limiting efficacy [12,15]. The molecularly targeted drugs, such as sorafenib, lenvatinib, cabozantinib, regorafenib, and ramucirumab, are multikinase inhibitors used as second-line options and have become the main therapeutic choices for advanced HCC patients [13,16,17,18]. Unfortunately, despite the proven survival extension in advanced-stage HCC, only ≈30% of patients responded to sorafenib. Moreover, this group developed resistance to sorafenib within 6 months [19]. Advances in immunotherapy have shown promise in HCC treatment, particularly with immune checkpoint inhibitors (ICIs). However, the response rate to ICIs as monotherapy is currently limited to 15–20% of HCC patients [20], while only 5.8% in CCA cases [21].

As the current management of hepatobiliary cancers based on the four pillars: surgery, radiotherapy, chemotherapy and immunotherapy remains unsatisfactory and represents a challenge for the multidisciplinary oncology team, there is an increased interest in developing new treatment modalities. Emerging, non-invasive, focused ultrasound (FUS)-mediated therapies based on high-intensity focused ultrasound (HIFU) and low-intensity focused ultrasound (LIFU) are cancer treatment modalities with promising results that could have the potential to positively influence some of the limitations of standard care. Advances in FUS medical technology have sparked increased interest from researchers and clinicians in less invasive treatment options that provide oncological control while reducing treatment-associated morbidity. Acoustically mediated, liver-targeted therapies such as HIFU thermal ablation and the innovative non-thermal ablation, histotripsy, together with the complex bioeffects of LIFU-based non-ablative modalities, are areas of current research and expanding clinical applications. The slow integration of new therapies together with the suboptimal uptake of surveillance for chronic liver diseases at risk of liver cancer development represent major gaps [6,7].

### Methods

We conducted a structured search of peer-reviewed studies indexed in PubMed, Web of Science, Scopus and Embase using the terms: “high-intensity focused ultrasound (HIFU)”, “histotripsy”, “low-intensity focused ultrasound (LIFU)”, “AND”/”OR” in conjunction with “hepatocellular carcinoma”, “liver metastases”, “biliary tract cancer”. Eligible publications included systematic reviews, meta-analyses, randomized and non-randomized trials, and prospective or retrospective clinical series evaluating FUS-mediated therapies used in hepatobiliary cancers. Internal industry-sponsored reports, corporate white papers, and advocacy foundation publications were excluded from the formal analysis. The significant heterogeneity of the study designs and the insufficiency of high-quality clinical data, especially those on the use of HIFU and histotripsy in biliary tract cancers, but also on the applications of LIFU in hepatobiliary cancers, did not make systematic review methodology feasible. Therefore, we adopted the narrative review format, which allows for an integrative analysis of the results of preclinical and clinical studies and their potential in the broader therapeutic context of these malignancies.

This work summarizes evidence from major scientific databases regarding the complex mechanisms, potential of current applications, limitations and challenges of FUS-mediated therapies in hepatobiliary cancers. It explores efficacy, safety and oncological outcomes of HIFU and histotripsy ablations and the non-ablative modalities using LIFU in the management of these difficult-to-treat cancers. Acoustically mediated applications will be addressed both as stand-alone alternative treatment options and in combination with conventional treatments, analyzing their potential synergistic effects. The existing gap in FUS-mediated applications in the treatment of biliary tract cancers, due to the lack of specific reporting of their inclusion in liver studies or their non-specific reporting as malignant biliary obstructions (MBO), leads to addressing them together with liver tumors.

## 2. Principle and Mechanism of FUS Relevant for Cancer Treatment

FUS principles are the same as conventional ultrasound (US) used in diagnostic imaging, but higher energy and intensity of acoustic waves are used in therapeutic purposes to induce the expected biological effects. HIFU was first proposed for therapeutic use in 1942, when Lynn et al. found it caused the destruction of liver tissue and changes in brain structure and behavior in living mice [22]. The use of therapeutic US for cancer treatment was established in 1979, when the potential of US to provide local tumor control and to enhance other treatment modalities was recognized [23]. In 1999, Gelet et al. reported the first clinical study of the use of HIFU for the treatment of local prostate cancer [24]. The application of FUS has expanded to destroy rat liver tissues [25], increase membrane permeability to enhance the uptake and cytotoxic effects of drugs, resulting in tumor cell death [26,27,28]. During the next decades, many researchers dedicated efforts to study, experiment and understand the mechanism of action of FUS beams on tumoral tissue. The lack of development of real-time guidance systems for tumor targeting and intra-procedural monitoring of FUS-assisted ablation has been a barrier to clinical translation. Technological advancement of medical imaging guidance accelerated the development of this new field of research and clinical applications.

FUS techniques use highly focused sound waves to precisely concentrate energy within a defined targeted tissue, avoiding damage to surrounding healthy areas. This allows highly localized energy deposition with millimeter-scale spatial resolution and deep tissue penetration [29]. Depending on the applied acoustic parameters, such as frequency and intensity, FUS can be divided into HIFU and LIFU [30]. HIFU is used in focal ablative techniques such as HIFU thermal ablation and recently developed non-thermal mechanical ablation, histotripsy. LIFU is the basis for non-ablative applications of FUS through the multiple bioeffects induced in human tissues. The chemical effects, microenvironmental regulation (facilitating drug delivery and immunomodulation) and the reversible blood–brain barrier opening [31,32], and blood-tumor barrier opening are studied in various combinations with standard cancer therapies targeting their efficacy enhancement.

### 2.1. Mechanism of the Ablative FUS-Mediated Treatment Modalities

#### 2.1.1. High-Intensity Focused Ultrasound Thermal Ablation (T-HIFU)

The thermal effects are generated by the large amplitude, high-duty cycle of FUS, and occur when the local tissue temperature rises higher than the level causing thermal necrosis (>56 °C) [33]. It is the result of acoustic energy absorbed by tissue and represents the basis of the T-HIFU technique [31]. Ultrasound-guided High-Intensity Focused Ultrasound (USgHIFU) and Magnetic Resonance-guided High-Intensity Focused Ultrasound (MRgHIFU) for mapping the tumor zone [34,35] increases its potential for precise tumor ablation. Advances in HIFU technology have increased its use in alternative oncological strategies for focal treatment of solid tumors and palliative pain relief in bone metastases [36,37]. HIFU thermo-ablation removes malignant tissues, without affecting healthy tissues, and the HIFU biological effects can be enhanced with microbubbles (MBs) or nanobubbles (NBs) [38].

#### 2.1.2. High-Intensity Focused Ultrasound Non-Thermal, Mechanical Ablation (M-HIFU), Histotripsy

The mechanical effect of FUS is harnessed in mechanical HIFU (M-HIFU), which uses acoustic energy to fragment cells and proteins through the effect of cavitation, micro-streaming and radiation force [39]. The cavitation effect consists of the formation and collapse of bubbles to mechanically disrupt tumor cell membranes and structures at lower temperatures. M-HIFU or cavitation cloud histotripsy uses very high pressure in combination with short-pulse ultrasound waves, which are in the range of microseconds, leading to mechanical destruction and liquefaction of the irradiated tissue [38]. Recently developed histotripsy is based on the controllable initiation of cavitation from endogenous nanoscale bubbles within tissues that induce a non-thermal tumor disruption [40]. Boiling histotripsy requires longer pulses, which are still in the range of microseconds and produce boiling bubbles, and lower peak pressures than cavitation cloud histotripsy [38]. Compared to HIFU thermal ablation, which uses continuous HIFU, histotripsy uses short pulses, low-duty cycle acoustic waves that create cavitation bubble clouds, selectively and precisely destroying target tissue while sparing critical structures such as bile ducts, ureters, and blood vessels. This greater precision of this technology is being exploited in treating tumors near vital structures. It is also being explored for its potential to induce immunomodulation and promote abscopal inhibition of distant, untreated tumors through CD8+ T-cell responses [41]. The necrotic, acellular debris resulting from histotripsy releases DNA, RNA and proteins, acting as neoantigens, which enhances antitumor immunity, dendritic cell migration and T-cell activation, making this procedure a potential adjuvant for immunotherapy.

### 2.2. Mechanism of LIFU Used in Non-Ablative FUS-Mediated Treatment Modalities

Low-intensity focused ultrasound (LIFU) utilizes lower energy levels to achieve therapeutic effects without immediately destroying the tissue at the focal spot.

LIFU-based non-ablative FUS methods are emerging as a non-invasive therapeutic modality being investigated as an adjuvant therapy in cancer treatment. Unlike HIFU, which ablates tumors via heat, LIFU uses low-intensity mechanical waves (typically <3 W/cm^2^) to produce minimal heat and primarily induce non-thermal effects [42]. It produces mild heating of the tumor (42–45 °C) and makes cancer cells more sensitive to other treatments like chemotherapy or radiation therapy, and stimulates anti-tumor immune response. LIFU mild heating modality has been explored for applications like thermal-controlled drug release and radiosensitization [43]. At this moderate temperature, cells do not undergo necrosis but instead activate a cellular stress response that includes the upregulation of heat shock proteins, an increase in cell membrane fluidity and produce transient vascular permeability [44]. These changes may facilitate the release and extravasation of tumor-derived antigen biomarkers into the circulation, providing a mechanistic basis for enhanced detection via sonobiopsy [45].

The sonoporation effect is a LIFU-based mechanism resulting in the formation of openings in the tumor vasculature walls, induced by US-triggered oscillations and destruction of microbubbles. It represents the mechanism behind enhanced delivery of therapeutics. Improvements in spatially confined delivery of chemotherapy and genes to target tumor tissues while reducing systemic dose and toxicity are based on the development of ultrasound and microbubble (USMB) mediated drug delivery technology [46].

In addition, LIFU can mechanically and temporarily open biological barriers, such as the blood–brain barrier [31] and blood-tumor barrier, enhancing the permeability of tumor blood vessels to allow targeted delivery of therapeutic agents (e.g., chemotherapy drugs: gemcitabine, cisplatin or nanoparticles) to penetrate deeper into solid tumors [47].

Advances in focused acoustic beam technology offer emerging FUS-based applications in difficult-to-treat liver and biliary tract malignancies, sparking increased interest from researchers and clinicians.

## 3. Applications of FUS-Mediated Techniques in Hepatobiliary Cancer Management

FUS-mediated cancer treatment modalities are versatile techniques with broad applications [45]. They can be used as a stand-alone, non-invasive treatment for selected localized solid tumors or in combination with conventional therapies to enhance their effectiveness. From a broad applicability perspective, the following sections address the current status of the use and potential of HIFU and LIFU in the management of hepatobiliary malignancies.

### 3.1. HIFU-Based Ablative Applications in Hepatobiliary Cancer Management

#### 3.1.1. HIFU Thermal Ablation of Hepatobiliary Tumors

Surgical resection and transplantation are the standard first-line treatments for early-stage HCC, but less than 10% of patients fulfilled preoperative criteria for resection [48]. Despite advances in surgical techniques, only 25% of patients with HCC in cirrhotic liver can be treated with surgical resection at diagnosis [49]. Novel liver-directed strategies are options to treat selected patients who are ineligible for surgical resection, and in some cases, they achieve curative intent. Catheter-directed and percutaneous locoregional interventions have evolved as treatment modalities for unresectable HCC [50]. HIFU thermal ablation is a clinically used and extensively studied FUS-based method for the removal of various solid tumors while preserving surrounding healthy tissues [38]. As an extracorporeal, noninvasive, precise focal ablation, without the need for surgical incision, it becomes particularly attractive for HCC treatment. HIFU ablation utilizes a frequency of continuous US wave, 0.8–3.5 MHz, which can be focused on at a distance from the therapeutic transducer, inducing coagulative necrosis by elevating the tumor tissue temperature to above 60 °C [51,52].

##### HIFU Potential as Stand-Alone FUS-Mediated Therapy of HCC Compared with Other Liver-Directed Therapies

Used as a stand-alone treatment, initial results of HIFU ablation for HCC management have been promising, with a complete ablation rate of 28.5–68% [52,53]. HIFU ablation of HCC has been shown to be safe, and intraprocedural treatment assessment provided an accurate measurement of the ablated zone and correlated well with MRI follow-up [54]. Given the fact that HCC is associated with cirrhosis in 80–90% of individuals [55], the lower limit of liver function and other patient factors that influence the tolerance of HIFU treatment were analyzed. The results have shown that HIFU is effective for the treatment of HCC patients with poor liver function, being well tolerated and safe for Child-Pugh A and B and selected Child-Pugh C patients considered ineligible for surgical resection. Patient age was the only factor that was found to be significant in HIFU intolerance [56].

HIFU has been used in HCC mainly as co-adjuvant therapy in sequential oncology protocols. However, there are some studies that have compared HIFU with RFA or TACE as single treatment modalities. A prospective clinical trial [57] directly compared HIFU applied as a sole strategy with RFA alone, in selected cases of recurrent HCC. It reported comparable results with no significant differences in terms of survival, and a tendency towards a better tolerance profile with HIFU and reduced side effects. HIFU alone vs. TACE alone in HCC patients was compared in two studies, and both reported a significantly higher rate of complete tumor ablation and higher survival, along with decreased length of hospital stay in the HIFU group [58,59]. In the study that included unresectable HCC patients [59], the rates of complete tumor response, partial tumor response, stable disease and progressive disease according to the modified Response Evaluation Criteria in Solid Tumors (m RECIST) were 50%, 7.7%, 25.6% and 7.7%, respectively, in the HIFU group. The TACE group had the corresponding rates at 0%, 21.2%, 63.5% and 15.4%, respectively. The 1-year, 3-year and 5-year survival rates were significantly higher in the HIFU group than in the TACE group. HIFU ablation demonstrated safety and efficacy as a method for unresectable HCC with a survival benefit over TACE applied stand-alone. Two years later, a state-of-the-art publication on HIFU applied to hepato-bilio-pancreatic cancers concluded that HIFU seems to add survival advantages over TACE alone and similar results when compared to RFA [60]. The role of HIFU in hepatobiliary malignancies was explored in a recent systematic review [61] reporting that of all HIFU treatment procedures analyzed in 24 studies, 55% resulted in complete tumor ablation [54,56,57,58,62,63,64,65,66,67,68,69,70,71,72,73], and 45% achieved only incomplete ablation. Technical success of HIFU is reported when complete tumor ablation is achieved, defined as the disappearance of the lesion contrast pattern and reduction in tumor blood supply, assessed at one week and one month post-procedure by CT/MRI imaging. In ten studies out of the 24 systematically reviewed, HIFU was used as an adjuvant or secondary treatment [57,62,70,72,73,74,75,76,77,78].

##### HIFU Thermal Ablation as Co-Adjuvant in Combination Treatment Strategies

Studies reporting HIFU as a co-adjuvant of TACE [76,79,80,81], including only one randomized trial [79], suggested that this combination therapy achieves better disease control as compared to TACE alone.

Some evidence suggests that HIFU combined with other loco-regional therapies, particularly TACE, is more effective than any single technique. The complete tumor ablation rate was higher, at 66%, in studies that combined HIFU with TACE, RFA, or PEI [61]. A significant survival advantage obtained by combining HIFU with TACE over TACE alone was shown in a recent meta-analysis [82]. Another meta-analysis [83] found that compared with TACE alone, HIFU combined with TACE had better efficacy, a higher survival rate, and a lower incidence of adverse reactions.

##### HIFU as FUS-Mediated Bridging Therapy in Liver Cancers

Various bridging therapies have been used to reduce the dropout rate in HCC patients awaiting liver transplantation, a treatment modality that may benefit a small number of cases due to the lack of liver grafts. Loco-regional therapies offer good alternatives to resection in these patients [84,85]. A bridging therapy must help to improve liver function and not cause further liver decompensation. TACE and RFA are the most widely used to decrease the dropout rate of liver transplant candidates. Despite treatment applied before liver transplantation, the dropout rate for TACE ranged from 15% to 35% in different studies [86,87]. In addition, TACE is contraindicated in patients with Child-Pugh C cirrhosis, main portal vein thrombosis, arteriovenous shunting, and extrahepatic metastasis [58]. RFA has recorded better results, but the dropout rate also ranged from 5.8% to 14% in various studies [88,89]. TACE and RFA are effective bridging therapies, but only for selected patients [90,91,92,93,94]. They are not safe for patients with liver insufficiency, thrombocytopenia and ascites.

There are some studies that analyzed the role of HIFU as bridging therapy in advanced liver malignancies [58,74,95]. The first study that used HIFU ablation as bridging therapy was successful for one HCC patient awaiting liver transplantation. Despite its extremely low platelet count (20 × 10^9^/L), the ablation was successful, maintaining similar liver function before and after HIFU and no adhesion was identified in the liver transplantation performed six months later [74]. The same research group continued to successfully use HIFU ablation as a treatment modality for waitlisted HCC patients, showing safety in advanced cirrhosis and gross ascites where RFA might be contraindicated [58]. HCC tumors located at sites considered difficult for percutaneous RFA were safely HIFU ablated in patients with poor liver function or decompensated cirrhosis, including ascites, advanced stage of disease at Child-Pugh B or above [61]. It was reported that three out of five patients who underwent HIFU ablation subsequently received transplantation, after a median waiting time of 9 months. The rest of the HIFU-treated patients who were still waiting for liver transplantation during the study period had stable disease without post-procedural complications. HIFU ablation was a successful bridging therapy in cases with TACE and RFA contraindications due to the poor liver reserve. It can potentially reduce the dropout rate of liver transplantation candidates and may prolong the survival of selected patients for whom transplantation is not an option. The HIFU procedure was safe; none of the patients developed liver failure after treatment [58].

In the presence of ascites or coagulopathy, HIFU could be the only possible option to keep a patient on the liver transplantation list or to treat HCC recurrences, as the other more invasive locoregional therapies (cryoblation, PEI, TACE and RFA) are contraindicated [60]. Moreover, it can be useful in areas where the liver donation rate is low by potentially reducing the dropout rate [96].

##### HIFU Application for Thermal Ablation of Liver Metastases

The incidence of LMs has been markedly increasing [97]. The dual blood supply of the liver by both the hepatic artery and the portal vein not only makes it uniquely susceptible to metastases from gastrointestinal cancers but also accessible to interventional therapies. Liver is a fertile ground for metastases that spread via blood circulation and may develop virtually from any organ [98]. Only 10% to 15% of LMs patients meet the clinical criteria for surgery [99,100] with a 5-year postoperative survival rate of only 30% [101]. An active treatment strategy for LMs, including loco-regional therapies, is required to delay disease progression and improve survival outcomes. HIFU is currently used for the ablation of LMs from colorectal and stomach cancers [102,103]. It was reported as a safe and feasible method of treating small (<3 cm) LMs. Reported results of early prospective clinical and feasibility studies on the treatment of LMs with HIFU are presented in Table 1.

A phase I clinical trial evaluated the safety and efficacy of US-guided HIFU (USgHIFU) in patients with CLM who had contraindications to resection and RFA. It was concluded that HIFU was safe, achieved a promising tumor response rate and should be considered in patients who are not eligible for other local therapies [104]. Compared with MWA, HIFU was associated with lower hospitalization costs, less trauma, and no serious postoperative complications [108]. The effectiveness and safety of USgHIFU ablation for 238 patients with CLM who were unsuitable for hepatectomy were analyzed in a multicenter retrospective study. Contrast-enhanced MR imaging and/or contrast-enhanced CT examinations were conducted, and mRECIST was used for the assessment of tumor ablation effectiveness before and after treatment, and every 3 months thereafter. The study demonstrated that USgHIFU is a feasible, safe and effective therapeutic option for surgical unresectable CLM patients without extrahepatic metastases [109]. HIFU ablation as an adjuvant to systemic chemotherapy in patients with inoperable CLM was retrospectively analyzed. The combination of HIFU with systemic chemotherapy was safe, significantly improved local control rates and prolonged median progression-free survival (mPFS), especially for patients with LMs < 5 cm [110]. The combination of USgHIFU ablation with immunotherapy was reported to be clinically feasible and safe for LMs treatment in a prospective non-randomized trial [107]. A sequential treatment strategy has recently been investigated, combining a targeted antiangiogenic therapy administered before HIFU treatment in patients with HCC and LMs. The study reported that the association of antiangiogenic agents improved the short-term efficacy of HIFU treatment in advanced primary and secondary liver cancer [111]. The clinical and immune effects of HIFU on CLM were evaluated, demonstrating a significant improvement in overall survival (OS), with a median survival of 52 months. This study also compared a subgroup of patients receiving HIFU combined with first-line therapy with patients receiving chemotherapy alone. OS was significantly improved in the HIFU + chemotherapy group compared to chemotherapy only. In addition, serum IL-6 levels post-HIFU treatment were significantly decreased, suggesting enhanced immune responses [112].

An intra-operative HIFU prototype with a powerful toroidal transducer was used in a phase I–IIa prospective study that represented the first clinical experience of intra-operative HIFU in patients with CLM. Early reports indicated that the phase I patients underwent successful superficial and deep HIFU ablations that were achieved with a precision of 1–2 mm. Intra-operative HIFU was feasible, safe and effective in ablating areas of the liver scheduled for resection. (ClinicalTrials.govNCT01489787) [105]. The next stage was an ablate-and-resect, prospective, single-center, phase II study, which attempted ablation of small metastases with a 5 mm margin, prior to planned hepatectomy (ClinicalTrials.govNCT01489787) [106]. In total, 23 of 24 CLMs were successfully treated (95.8%), and no damage occurred to extrahepatic tissues. Study concluded that intra-operative HIFU can safely and accurately produce large ablations with real-time guidance prior to surgical resection.

The results of retrospective and non-randomized comparative clinical studies of HIFU for liver metastases are presented in Table 2.

##### Quality of Evidence from Studies on the HIFU Ablation of Liver Metastases

**Figure 1:**
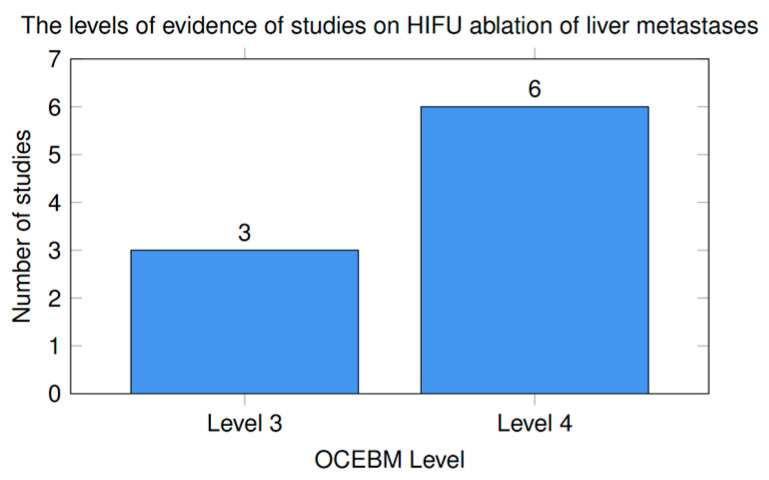
The levels of evidence of studies on the HIFU ablation of liver metastases.

These studies are predominantly Level 4 evidence, indicating that current clinical evidence for HIFU in liver metastases is still mainly based on: early-phase trials, case series, observational cohorts, and feasibility studies. Only a few studies reach Level 3, mainly due to comparative or matched cohort designs. The levels of evidence of studies included in Table 1 and Table 2 are represented in Figure 1.

As an early-phase single-arm study, the phase I trial, Yang et al. [104], was not designed to establish comparative efficacy. The studies with intra-operative HIFU [105,106] provide valuable proof-of-concept data, but their small sample sizes, highly selected surgical populations, and surrogate endpoints limit external validity and oncologic interpretation. Although the study combining USgHIFU with immunotherapy [107] is of interest, the lack of randomization and modest cohort size prevent firm conclusions regarding the additive clinical benefit. The multicenter retrospective series of 238 patients with unresectable CLM [109] is one of the larger available cohorts. However, the retrospective design introduces risks of selection bias and variability in patient selection and follow-up. As a retrospective analysis based on propensity score, the evaluation of HIFU combined with systemic therapy [110] improves comparability, but there is the possibility of residual errors, and such analyses cannot substitute for RCTs. The findings of the retrospective studies that have explored multimodality strategies [111,112] are hypothesis-generating but require prospective validation. The currently available clinical evidence for HIFU in liver metastases is heterogeneous and consists predominantly of early-phase feasibility studies, retrospective observational cohorts, and non-randomized comparative analyses. Therefore, conclusions regarding efficacy should be interpreted cautiously, as the evidence base remains less mature than for established ablative modalities such as RFA and MWA.

##### HIFU Potential in the Management of Biliary Tract Tumors

Biliary tract cancers, including cholangiocarcinoma (CCA) and gallbladder cancer, frequently present with malignant biliary obstruction (MBO). While surgical resection remains the only curative intent, the majority of patients present with unresectable disease due to anatomical constraints or advanced stage. In these cases, loco-regional therapies are vital for palliation and local disease control. Loco-regional therapies for patients with unresectable intrahepatic CCA include radiation therapy, TACE, transarterial chemoinfusion, radioembolization and RFA [113,114]. Clinical evidence on HIFU potential in the treatment of biliary tree malignancies is limited and heterogeneous [60,115,116].

##### Quality of Evidence from Studies on HIFU Potential in the Management of Biliary Tract Tumors

**Figure 2:**
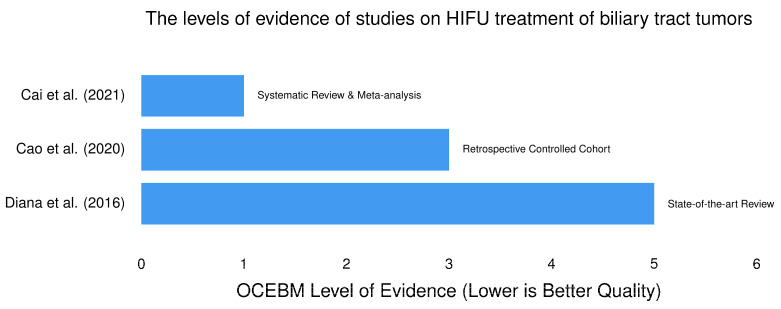
The levels of evidence of studies on HIFU treatment of biliary tract tumors [60,115,116].

The levels of evidence of studies on HIFU treatment of biliary tract tumors are presented in Figure 2.

Cao et al. [115] reported that unilateral stent insertion followed by HIFU is safe and effective for high-grade hilar obstructions. Recent high-level evidence supports the integration of HIFU with conventional stenting. A systematic review and meta-analysis [116] of six studies involving 429 patients with MBO demonstrated that the combination of biliary stenting and HIFU ablation significantly outperformed stent insertion alone. The thermal effect of HIFU appears to delay tumor ingrowth and overgrowth, extending the duration of biliary drainage. The combined approach resulted in improved OS, suggesting that local tumor debulking in the biliary tree has systemic benefits by reducing cholestasis-related complications. Retrospective data specifically targeting hilar cholangiocarcinoma have shown the feasibility of this “double-hit” approach. Clinical series highlighted that HIFU can be used to stabilize patients or reduce tumor burden while awaiting further systemic treatment and to reduce the intraductal tumor mass, thereby improving the quality of life by mitigating jaundice and pruritus [60]. While external beam HIFU is the current standard, the field is shifting toward more targeted delivery methods using intraductal transducers. Unlike extracorporeal HIFU, new investigational interstitial/intraductal transducers are being developed to be used endoscopically or percutaneously. This allows for direct thermal ablation of the CCA from within the bile duct, potentially reducing damage to surrounding sensitive structures like the duodenum or portal vein [60]. Investigational frameworks suggest that HIFU-induced hyperthermia may increase the permeability of the desmoplastic stroma characteristic of CCA, potentially enhancing the delivery of systemic chemotherapy to the tumor site. Despite promising data [115,116], the adoption of HIFU in biliary tract tumor management remains limited by the lack of large-scale, disease-specific prospective RCTs. Future research must focus on standardizing ablation protocols and defining the optimal timing of HIFU relative to stent placement and systemic chemotherapy. There are few studies on other loco-regional treatments such as RFA in patients with intrahepatic cholangiocarcinoma and they have reported less optimal results than those observed in the treatment of HCC. To establish first-line local-regional therapeutic options for patients with unresectable intrahepatic cholangiocarcinoma, RCTs are recommended [117].

##### Potential Benefits of HIFU Compared with Other Thermal Ablation Techniques in Liver Cancers

Unlike RFA or MWA, where blood flowing in nearby vessels carries away the heat and prevents complete tumor ablation, HIFU delivers energy rapidly enough to raise temperatures above 70 °C and destroy tumor cells before blood flow dissipates the heat. Some potential benefits of HIFU compared with RFA and MWA in liver cancer treatment are presented in Table 3.

Non-invasiveness, non-ionization, reduced post-procedural morbidity [96,118], and short recovery are benefits of HIFU compared to conventional cancer treatments.

As there is no upper limit of tissue tolerance to repeated HIFU exposure [34], it has the benefit of being a repeatable ablative intervention when necessary. However, a single MWA session might take 10 min, while a complex HIFU session can take hours under general anesthesia, which has its own risks. In addition, the cost-effectiveness trend and time spent on the operating table should not be ignored. Regarding repeatability, in cases of recurrence of HCC after initial ablation, RFA sessions, as HIFU ablation can be repeated, especially in patients in whom surgery is not feasible. Overall, HIFU is a well-tolerated thermal ablation of liver cancer, offering an alternative option to the patients previously thought to be untreatable surgically due to Child–Pugh B or C disease [56]. HIFU is suitable for lesions that are otherwise considered untreatable by RFA or MWA due to their proximity to large blood vessels or bile ducts [119]. The main benefit of HIFU over RFA and MWA is that it does not require puncturing the tumor, thereby avoiding the risk of bleeding or seeding of tumor cells along the needle tract [120]. However, the risk of needle tract seeding after RFA for liver tumors is low, in the range of 0–1.1%, with a similarly low risk of 0.47% for MWA.

This can be minimized by avoiding pre-ablation biopsies when the diagnosis can be made by imaging and using a coaxial sheath to reduce contact of the therapeutic needle with healthy tissue during removal.

It has lower procedural bleeding, bile duct leakage and infection risk than RFA and MWA, because the skin and liver capsule are not punctured [96,121]. HIFU ablation provides a feasible therapy for HCC in patients with advanced cirrhosis and the elderly [66]. In patients with gross ascites, HIFU treatment can be performed safely as the ascites inside the peritoneal cavity not only provides a clear image for the diagnostic US probe but also serves as a good medium for energy transfer. Moreover, the existence of intraperitoneal fluid protects the subcutaneous tissue from damage by FUS energy [56]. Unlike RFA, in HIFU, the temperature at the off-target zone remains static, thus sparing healthy tissue from thermal damage. In addition, patients with hyperbilirubinemia, low serum albumin level and thrombocytopenia showed intolerance to RFA but tolerated HIFU [122]. HIFU is a potential treatment option for tumors in high-risk locations (e.g., hilum or near the gallbladder) where the mechanical trauma of an MWA antenna could cause damage. It had fewer serious complications compared to MWA, which involves, in some studies, a 7% rate of biliary leakage (0% in corresponding HIFU studies) [108]. In a recent retrospective study, overall survival and disease-free survival rates of patients treated with HIFU were comparable to those with RFA or MWA. Its benefit is that it is well tolerated in HCC patients with advanced cirrhosis, ascites and coagulopathy, ineligible for other ablative means, serving as a therapy of last resort [123]. These potential benefits of HIFU must, however, be viewed with caution and in the context of the patients treated. There is considerable overlap in the clinical application of liver-directed therapies, their selection being related to the type, number and location of tumors and the patient’s comorbid status. While HIFU and RFA were comparable in terms of effectiveness and safety in the treatment of HCC [60], current evidence is limited, and more prospective RCTs are warranted to confirm these findings.

Although unlike HIFU, MWA provides higher intratumoral temperatures, faster treatment times, and is suitable for treating larger tumors (>5 cm), its application is restricted to sensitive areas, such as the hepatic hilum, bile ducts, and head of the pancreas, to avoid accidental injury. HIFU is highly effective for small tumors (<3 cm), but its efficacy drops off significantly for larger masses compared to MWA, which can create larger, faster ablation zones.

##### Quality of Evidence from Studies on the Potential Benefit of HIFU Compared cu RFA and MWA in the Treatment of HCC

**Figure 3:**
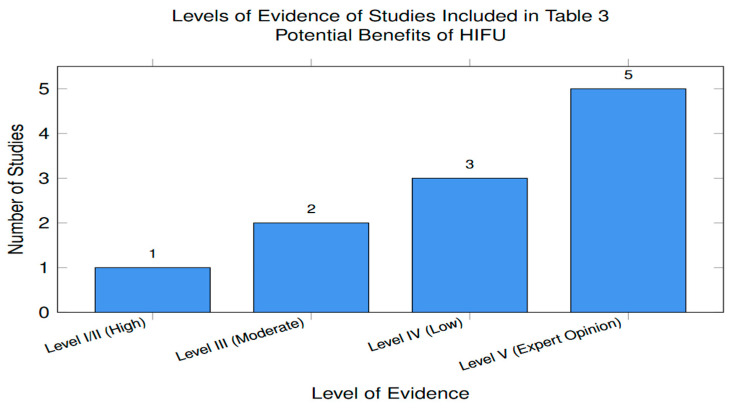
Representation of the levels of evidence of studies included in Table 3.

The levels of evidence of studies included in Table 3 are represented in Figure 3.

Classified according to the Oxford Center for Evidence-Based Medicine (OCEBM) hierarchy, the majority of studies (8 of the 11 papers) consist of Level IV or V evidence. This distribution is typical for an emerging technology such as HIFU, where case series and expert reviews (Level IV/V) form the initial knowledge base before higher-level studies are conducted. Although case series (Level IV) and narrative reviews (Level V) are foundational and the necessary starting point for emerging therapies, they lack the validation of large-scale RCTs directly comparing HIFU with RFA/MWA for long-term survival. Furthermore, they carry the risk of bias, requiring more Level I evidence (systematic reviews and RCTs) for definitive clinical proof. Some studies (e.g., Ng KK 2011, Tsang SH 2021) [66,119] may have potential selection bias because they are single-center experiences. Patients with HCC selected for HIFU often have specific characteristics (e.g., unsuitable for invasive probes) that differ from the much larger number of patients treated with RFA and MWA, making direct comparison difficult. Until more systematic level I reviews (such as the Sehmbi 2021 study) [61] or RCTs are conducted, the potential benefits of HIFU should be considered only promising. Furthermore, only head-to-head RCTs can determine whether an emerging therapy is superior or non-inferior to the current standard of care.

##### Limitations of the HIFU Thermal Ablation

HIFU ablation has some limitations. It is a time-consuming procedure [120], as elementary ablations are small and must be juxtaposed to treat tumors [106]. HIFU equipment availability is only in some centers, the high cost, especially when MRI is used as guidance, and the requirement of general or epidural anesthesia are other factors that limit the clinical applicability of this procedure [120].

##### Disadvantages of HIFU Thermal Ablation

Disadvantages of HIFU are the pre-HIFU surgical rib removal and intra-pleural infusion of warm saline solution, which are still used to obtain a favorable acoustic therapeutic windowing in order to specifically treat liver tumors located in difficult positions (e.g., the liver dome) that are obstructed by the rib cage. Although effective, those methods drastically reduce the appeal of HIFU in terms of minimal invasiveness [75]. Alternative techniques were developed to avoid these invasive steps, such as using specialized transducers (e.g., segmented transducers) to steer around ribs, placing degassed water-filled balloons to displace surrounding tissue, or relying on patient breath-holding controlled by the anesthesiologist during the procedure [63,75].

The common post-HIFU complications reported were those at the application site: skin burns, local pain and fever (2%). Serious complications reported in a minority of HCC patients treated with HIFU include rib fractures, pneumothorax, pleural effusion, biliary obstruction, and fistula formation [124]. HIFU procedure-related deaths were not reported [61]. Patient age was the only factor found to be significant in HIFU intolerance.

##### Challenges and Technical Limitations of HIFU Thermal Ablation

HIFU treatment of tumors situated in the liver dome, close to the rib cage or in close proximity to the stomach, bowel, gallbladder, heart, major blood vessels or bile ducts presents unique challenges. However, meticulous pre-procedural preparation and special maneuvers during HIFU can achieve complete ablation with few complications [119,125]. Several physiological and technical limitations that challenge the widespread adoption of HIFU for liver cancer treatments and potential solutions are presented in Table 4.

The respiratory movement affects the precision and efficiency of HIFU liver tumor ablation [126]. A solution is to have the patient undergo ventilator-controlled breath-holds while under general anesthesia [127]. Ultrasound-guided HIFU has been shown to be able to track the 3D motion, overcoming this challenge. Artificial ascites or hydrodissection is an effective technique for safe ablation of hepatic dome HCC [128,129]. It creates a separation of the hepatic dome from the diaphragm, thereby preventing damage to the diaphragm and pleura during thermal ablation. Artificial pleural effusion using saline is a valuable adjunctive technique, creating a safe percutaneous path or good sonographic window when US is used for image guidance [125]. A pre-procedural preparation is the surgical removal of the 7th to 9th ribs 2 weeks prior to HIFU for ablation of larger HCC on the right lobe, partially obscured by the rib cage [119]. The presence of fast-flowing blood vessels adjacent to the ablation target tissue may reduce the efficacy of ablation due to the heat-sink effect. Combining TACE with HIFU reduces the “heat sink” effect in liver cancer treatment by decreasing blood flow to the tumor, facilitating larger ablation zones and complete necrosis [83].

The place of HIFU in the hepatobiliary cancers management algorithm remains to be defined [60]. Although it is a promising ablation technique, the available clinical evidence for HIFU in hepatobiliary malignancies is heterogeneous. It consists predominantly of early-stage feasibility studies, retrospective observational cohorts, and non-randomized comparative analyses, and lacks the validation of large-scale RCTs, compared to established techniques. Therefore, conclusions regarding efficacy should be interpreted with caution, as the evidence base remains less mature than for conventional ablative modalities.

### 3.2. Histotripsy, the First Non-Thermal HIFU Ablation of Hepatobiliary Tumors

Histotripsy is an emerging treatment that uses focused sound waves to disintegrate tissue [130], creating microbubbles inside the cytoplasm of treated cells, which leads to mechanical tumor disruption. Applied for tumor ablation, histotripsy liquefies the target tissue into an acellular homogenate, and the remains are absorbed by the body within 1–2 months, leaving small scars. US imaging is used to guide histotripsy during treatment, as cavitation can be visualized on B-mode ultrasound as a hyperechoic area, and MRI is used to follow up post-treatment effects. The precision of liver tumor ablation by histotripsy was first demonstrated in rodent HCC models, where it consistently reached submillimeter transition zones and significantly slowed tumor progression compared to the control group [131]. Beyond its ability to destroy tumors, preclinical studies have indicated the possibility that tumor ablation by histotripsy may also produce systemic antitumor immune effects. This led to a much greater release of intact immunogenic tumor antigen and intratumoral infiltration of CD8+ T cells than RFA or radiation [132]. The release of tumoral antigens stimulates the innate and adaptive immune system to recognize and attack remaining tumor cells, potentially leading to an abscopal immune response where distant tumor sites are positively affected. Another preclinical study found that histotripsy releases immune-stimulating molecules at magnitudes superior to thermal ablation modalities, increasing innate immune system activation in vivo [133]. Murine model studies [132,134] demonstrated that histotripsy enhanced the infiltration of CD4+ and CD8+ T cells and elevated IFN-γ levels, even inducing abscopal effects when combined with immune checkpoint inhibitors.

These findings have begun to be translated into clinical studies, when a liver histotripsy-mediated abscopal effect was reported. This appeared in the form of volume reduction in the nontreated tumor lesions in the same organ, as well as sustained reduction in tumor marker [carcinoembryonic antigen (CEA)] and increased serum IFN-γ and TNF-α after liver histotripsy [135]. A potential synergy between histotripsy and immunotherapies in cancer treatment was suggested.

Early clinical trials have demonstrated that histotripsy is safe and effective, particularly in liver and kidney tumors [130]. The clinical studies that reported the safety and efficacy of liver tumor histotripsy are presented in Table 5.

Technical success in these trials is defined as achieving the planned ablation volume with complete tumor coverage on contrast imaging (typically MRI or CT) within the protocol timepoint (e.g., ≤36 h post procedure). Technique efficacy at 30 days post procedure is a separate reported measure of persistent absence of residual enhancing tumor by contrast-enhanced MRI scan at 1 month.

First-in-man histotripsy ablation of hepatic tumors was performed in a multicenter phase I trial (Theresa feasibility study) on 8 patients (1 HCC, 7 LMs), all presenting unresectable end-stage multifocal disease. It achieved complete technical success with median volume reductions of 40% to 70% within three months. Even tumors adjacent to critical hepatic structures were safely ablated. It has shown early promises, demonstrating short-term efficacy and no procedure-related complications [136].

A variety of advanced liver cancers have been treated [141] for downstaging, bridging surgery or transplantation [142] or palliation [138].

The findings of the Theresa feasibility study were confirmed by a global safety analysis that analyzed 230 hepatic histotripsises of primary and secondary tumors [140]. It provided initial post-FDA approval data, demonstrating its safety profile comparable to existing liver-directed therapies and reported no major device-related complications.

The feasibility and safety of histotripsy across diverse liver tumor contexts were established in the next clinical trials. The parallel United States and European Union and England #HOPE4LIVER trials were prospective, multicenter, single-arm studies that enrolled 44 patients (41% had HCC and 59 had LMs) with a maximum pretreatment tumor diameter of 1.5 ± 0.6 cm. The technical success, defined as achieving the planned ablation volume with complete tumor coverage on MRI contrast imaging at ≤36 h postprocedure, reached 95%. This result compares favorably with reported rates of RFA and MWA, where the rates in larger series have ranged from 48% to 95% across tumor types [143,144,145]. The technique efficacy at 30 days, defined as persistent absence of residual enhancing tumor by contrast-enhanced MRI scan at 1 month, was 83% and reported procedure-related major complications were 7%, both meeting the prespecified performance goals [137]. This was achieved in a single procedure and was comparable with those achieved in the earliest reports of RFA [146] and may improve with procedural experience [137]. The complication rate was within reported ranges for established thermal ablation (i.e., between 2% and 11% for the largest series of RFA and MWA) [144,147,148]. However, without head-to-head data, no definitive comparison can be made. Future studies should incorporate comparative designs to compare histotripsy ablation of liver tumors with existing locoregional therapies.

A retrospective study [138] evaluated the response to histotripsy, assessing computed tomography (CT) findings in the first three post-procedural months. A high percentage of patients (87.5%) showed no tumor growth within 3 months post-procedure, allowing some patients to be successfully downstaged for liver transplant or resection. In addition, lesion size decreased in tumors not targeted by histotripsy in four patients, including one palliative patient unresponsive to multiple prior treatments. It was reported to have a favorable safety profile, with a very low rate of major complications (<2%), making it comparable to other liver-directed therapies.

It was concluded as having potential for liver tumors ablation, achieving high rates of short-term oncologic local control while offering vascular and biliary tract protection advantages, unlike thermal methods. In combination with systemic therapies, it could aid in bridging or downstaging patients with advanced disease to achieve surgical resection or transplantation.

The 1-year clinical outcomes of patients enrolled in the #HOPE4LIVER trial of hepatic histotripsy were recently published in the first study that evaluated safety and efficacy outcomes beyond 8 weeks [139]. The reported local control rates of histotripsy ablated liver tumors at 1 year were consistent with current locoregional therapies, and the safety profile was favorable. The overall survival at 1 year was 73.3% for patients with HCC and 48.6% for patients with LMs, comparable with other liver-directed therapies for similar disease stages. These results over one year demonstrate the efficacy and safety of histotripsy in a real-world setting. Histotripsy liver ablations performed within the #HOPE4LIVER trial also demonstrated limited acute toxicities and few delayed toxicities, supporting this FUS-mediated therapy for patients with primary and secondary hepatic cancers [139].

#### 3.2.1. Quality of Evidence from Studies on Histotripsy Potential in the Treatment of Liver Tumors

**Figure 4:**
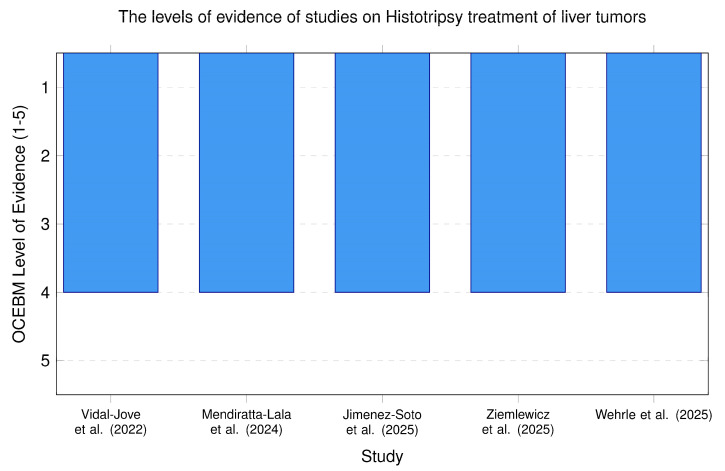
Representation of the levels of evidence of studies on hepatic tumor ablation by histotripsy [136,137,138,139,140].

The levels of evidence of studies on histotripsy ablation of liver tumors are represented in Figure 4.

Based on the Oxford Center for Evidence-Based Medicine (OCEBM) 2011 hierarchy, the studies reviewed on histotripsy treatment of liver tumors are classified as Level 4 evidence. This classification is primarily because these studies consist of feasibility trials, single-arm pivotal trials, and clinical case series, which lack a concurrent control group, leading to cautious interpretation of the results.

#### 3.2.2. Histotripsy Application as Bridging Therapy

Studies indicate that in some cases, patients who were previously ineligible for surgery or transplant became candidates after undergoing histotripsy. The first use of histotripsy as bridging therapy prior to liver transplant for HCC was reported on a patient with metabolic-associated steatotic liver disease (MASLD) and cirrhosis. Histopathology analysis of the explanted liver showed total necrosis of the treated area, with no residual viable tissue tumor, demonstrating the potential of histotripsy as an effective bridging therapy [142]. Another study reported recently that after histotripsy, three patients were successfully downstaged for liver resection and five were eligible for liver transplantation [138].

#### 3.2.3. Histotripsy Application for the Management of Biliary Tract Cancers

Biliary tract malignancies, particularly cholangiocarcinoma (CCA), are characterized by an abundant desmoplastic stroma—a dense, fibrous extracellular matrix that poses a significant challenge to conventional ablation. Unlike the softer, cellular architecture of liver cancer, CCA’s high collagen content and myofibroblast activity create a physical barrier that resists mechanical fractionation. Due to the high fibrous content, they are more resistant to cavitation than HCC tissue, necessitating specialized histotripsy parameters. Studies investigating histotripsy for the treatment of CCA are primarily found in preclinical research focusing on its feasibility due to the dense, fibrotic stromal components. Research using ex vivo dosimetry [149] has established that while HCC may be successfully ablated with standard parameters, CCA requires a significantly higher energy density. These preclinical investigations have quantified this resistance, revealing that the intrinsic threshold for cavitation in CCA is significantly higher than in liver tissue. It is a foundational preclinical study [149] that included in vivo experiments in a patient-derived xenograft mouse model, indicating the effectiveness of histotripsy in ablating CCA tumors, and ex vivo experiments comparing the histotripsy doses needed, highlighting the higher doses (250–1000 pulses/point) required for CCA tumors compared to the significantly lower doses effective for HCC or CLM tumors. This “stromal resistance” implies that histotripsy for biliary cancers requires tumor-specific pulse sequences to overcome the mechanical stiffness of the fibrotic matrix. While clinical data remains in infancy, the therapeutic window for histotripsy in biliary tract cancers is expanding.

The single human application of histotripsy in CCA treatment is a recently published case report on a 77-year-old woman with recurrent hilar cholangiocarcinoma with progressive biliary obstruction despite chemotherapy and irreversible electroporation. After 2 staged histotripsy treatments targeting tumors in the left and right biliary system, bilirubin normalized within 72 h, and the procedure was well-tolerated, with no complications. Furthermore, imaging evaluation at 6 months showed stable disease and maintained biliary patency, requiring only one stent exchange and highlighting the potential role of histotripsy to alleviate biliary obstruction and control local disease [150]. However, since the #HOPE4LIVER trial specifically excluded hilar and intrahepatic CCA, the evidence for histotripsy treatment of biliary cancers will come from independent case reports and the post-approval, multicenter studies. For patients with obstructive jaundice where stents have failed or are technically unfeasible, histotripsy offers a “mechanical debulking” that is non-thermal, thereby sparing the critical bile duct scaffolding while liquefying the intraluminal or compressive tumor mass [151]. Clinical implementation of histotripsy in CCA treatment is limited by the acoustic window. The proximity of the ribs and the presence of prior biliary stents can cause acoustic shadowing or reflection, requiring advanced transducer positioning or the use of water-path coupling to ensure the “bubble cloud” is accurately focused on the biliary lesion. Current research into intraductal or interstitial transducers for the histotripsy treatment of biliary tract cancers is extremely limited and remains largely in the early conceptual or preclinical phase. One of the primary drivers for developing intraductal histotripsy would be its non-thermal nature. Unlike thermal techniques, histotripsy spares collagen-rich structures like the bile duct scaffolding itself while liquefying the cellular tumor mass [151].

Preclinical advances are promising, suggesting that histotripsy has the potential to be used for the ablation of CCA while also highlighting the need for tumor-specific treatment parameters. While the pivotal #HOPE4LIVER trial demonstrated success in HCC and metastases, biliary tract cancers represent an emerging frontier currently supported by case reports. High-quality, disease-specific prospective trials with standardized protocols are required to validate the potential of histotripsy in the management of biliary tract cancers.

#### 3.2.4. Potential Benefits of Histotripsy

Histotripsy is emerging as a new liver tumor ablation technique because its unique mechanism has proven advantageous for tumors in difficult locations, where the heat-sink effect limits heat-based ablations. It has the ability to preserve critical vascular and biliary duct structures and stimulate innate and adaptive immune responses [130]. Unlike HIFU thermal ablation, histotripsy has specific clinical advantages: diminished heat sink effects resulting in lesions with sharp margins and effective removal of the treated tissue [151]. In addition, it presents evolving immunomodulatory potential. It has been shown to stimulate potent local and systemic immune responses and enhance the efficacy of immunotherapy [152,153].

The abscopal immune response induced by histotripsy, observed in preclinical studies and in the THERESA human trial, indicates its potential for use to improve immunotherapy in the treatment of non-targeted tumors. The immunostimulatory effect observed in the treatment of non-immunogenic cancers, such as liver cancer, is of current research interest [151]. Furthermore, its compatibility with systemic therapies was reported in a study performed in a center that utilized liver tumor histotripsy. The absence of interaction between histotripsy and chemotherapy or targeted agents allows it to complement ongoing systemic therapies, especially in advanced or multifocal liver tumors, synergistically maximizing treatment efficacy. All scheduled systemic medications throughout the peri-procedural period, including chemo/immunotherapy and anticoagulation, were continued, without bleeding events reported [141].

#### 3.2.5. Histotripsy as a Potential Theranostic Tool in Liver Cancer Management

Histotripsy can facilitate liquid biopsy. Bubble cavitation during histotripsy mechanically disrupts cell membranes and subcellular structures, releasing intracellular proteins, nucleic acids, and other cellular components into the circulation. These cellular biomarkers, such as cell-free circulating nucleic acids—microRNAs (miRNAs), messenger RNA, and DNA—that are very tissue specific and frequently dysregulated in many cancers, can be detected through integration of histotripsy with liquid biopsy (blood-based sampling) [154].

The cavitation-enhanced release of free nucleic acids has the potential to be used as a diagnostic tool for tumors in locations difficult to biopsy with a needle or as a therapy guidance tool to obtain data on the genetic content of a tumor during its ablation by histotripsy. This could contribute to combining chemotherapy or immunotherapy with histotripsy [151].

#### 3.2.6. Limitations and Contraindications for Histotripsy

Histotripsy has some limitations in its application to hepatic malignancies. It is most effective in soft, homogeneous tissue. In cirrhotic livers or tumors with fibrotic or calcified regions, bubble cloud formation and energy deposition become inadequate [130]. A preclinical study on a porcine model found reduced effectiveness of histotripsy compared to MWA in fibrotic tissue zones, highlighting the need for tailored treatment algorithms [155].

In addition, although it achieves submillimeter precision in targeting liver tumors, the efficacy diminishes with increasing tissue depth due to attenuation and aberration of the FUS beam. Advanced targeting systems utilizing phased arrays and AI-assisted aberration correction, utilizing feedback from cavitation emissions or MRI guidance, have demonstrated an up to 80% improvement in focal precision during transcostal liver ablation [156].

There are some contraindications for histotripsy. One is obesity by excessive subcutaneous fat that causes acoustic aberration, weakening the focal pressure below the threshold and affecting the completeness of ablation. If the lungs or ribs completely block the acoustic window, liver tumors in segment 7/8 are difficult to approach. Interposition of an intestinal loop in the path of the ultrasound beam causes interruption of histotripsy to avoid intestinal perforation. Although heterogeneity of studies is expected in the early period of adoption of new technologies, the lack of direct comparisons of data with other liver ablation modalities, the absence of long-term outcomes and higher-level evidence allows the interpretation of the results of available studies only as promising.

### 3.3. Low-Intensity Focused Ultrasound (LIFU) Applications in Hepatobiliary Cancer Treatment

Low-intensity Focused Ultrasound (LIFU)-mediated therapies are emerging non-invasive technologies based on their mechanical effects, such as sonoporation or sonopermeation, mild hyperthermia, targeted drug delivery, microbubble-mediated cavitation, sonodynamic therapy (SDT), and sono-immunotherapy. They can enhance the therapeutic efficacy of standard cancer treatments while minimizing damage to healthy liver tissue. Sonopermeation improves therapeutic efficacy by facilitating the penetration of therapeutic agents into cells. In addition to the formation of transient pores, sonopermeation alters tumor perfusion and affects the TME through its influence on tumor cells and surrounding tissues. LIFU sonoporation is a method of local drug delivery that has been applied in medical research and has proven its non-invasiveness, local applicability and safety. It restricts drug release to a focal US zone and enhances the effect of chemotherapy in the sonoporated areas [157,158]. The mechanism of increasing sensitivity to chemotherapy by sonoporation is based on the cavitation effect and the mechanical effect of LIFU [159,160].

The combination of LIFU with microbubbles (MBs), microbubble-assisted ultrasound (MBB-US), is used to increase vascular permeability for facilitating drug extravasation and tumor cellular uptake, normalize TME and restore tumor perfusion [161]. The use of MBB-US to deliver chemotherapeutic agents is referred to as sono-chemotherapy. Because the liver is a large, blood-rich organ, LIFU is often used to overcome the “washout” effect of systemic chemotherapy by releasing the drug only within the target focal zone. Most data regarding LIFU-based treatment of hepatobiliary malignancies remain preclinical. The applications and efficacy of LIFU in hepatobiliary cancers reported in preclinical studies are summarized in Table 6.

The applications and efficacy of LIFU in hepatobiliary cancers reported in clinical studies are summarized in Table 7.

#### 3.3.1. Sono-Chemotherapy in Hepatobiliary Cancers

Several preclinical studies [162,163,173,174] obtained promising results with LIFU combined with MB, increasing intra-tumoral drug uptake and inducing tumor volume reduction.

LIFU triggers the release of chemotherapy from microbubble and nanoparticle complexes by inducing cavitation, which enhances drug accumulation and penetration into tumors. This technique, known as ultrasound-targeted microbubble destruction (UTMD), improves the therapeutic efficacy of agents like adriamycin while reducing systemic toxicity in models such as VX2 liver cancer.

The antitumor effects of LIFU-mediated localized drug delivery of adriamycin-microbubble-PLGA nanoparticle complexes (ADM-NMCs) were studied on rabbits with VX2 liver tumor [162]. The highest volume inhibition rate (VIR) of tumor progression and apoptotic index (AI), and the lowest proliferating index (PI) were found in the ADM-NMCs combined with the LIFU group. This novel process for liver tumor targeting chemotherapy could delay tumor proliferation and accelerate apoptosis. An albumin-doxorubicin nanoparticle conjugated microbubble (ADMB) was developed to enhance the therapeutic efficiency of chemotherapy by sonoporation under US exposure [163].The doxorubicin loading efficiency was 82.7%, tumor growth was reduced by five times, compared to the control group, and liver toxicity was comparable to that of conventional therapies. The study concluded that tumor suppression can be maximized by combining LIFU exposure with intra-arterial ADMB administration.

A first clinical study using sono-chemotherapy [175] treated five patients with pancreatic adenocarcinoma using a combination of US and MBs following standard chemotherapy treatment with gemcitabine. The maximum tumor diameter decreased, prolonging the quality of life in patients treated with this combination compared to chemotherapy alone. The efficacy of LIFU sonopermeation was investigated in other clinical studies, showing that LIFU and MBs enhance the effects of conventional chemotherapy in patients with pancreatic cancer and liver metastases from digestive system tumors [164,170,176,177]. A randomized clinical study compared patients with colorectal liver metastases (CLM) treated with a combination of LIFU + MBs + chemotherapy with those receiving chemotherapy only [177]. The results showed that the LIFU + MBs combination is safe and feasible. Despite the considerable variation in treatment response between metastatic lesions, there was a tendency toward larger-volume reduction in lesions treated with FUS + MBs compared with control lesions. The study concluded that this is an available strategy for improving the effect of chemotherapy in CLM, but further multicenter trials with standardized protocols are required.

Cancers with hypoxic TME, such as biliary tract malignancies, present limited drug penetration and therapeutic resistance. Low-intensity pulsed ultrasound (LIPUS) was used to enhance drug delivery into the hypoxic regions of the TME in cholangiocarcinoma (CCA) [164].The antitumor efficacy of LIPUS-assisted chemotherapy was evaluated in a CCA xenograft mouse model. The penetration of gemcitabine and cisplatin was significantly enhanced, resulting in a fivefold increase in apoptotic cancer cell death and a significant reduction in CCA growth. LIPUS adjuvant therapy offers a safe and innovative therapeutic strategy for CCA.

The phase I single-center study TARDOX (ClinicalTrials.gov: NCT02181075) [171] enrolled patients with unresectable and non-ablatable primary or secondary liver tumors, aiming to assess the safety and feasibility of targeted drug release and enhanced delivery of lyso-thermosensitive liposomal doxorubicin (LTLD) triggered by mild hyperthermia induced by LIFU. It concluded that this combination treatment modality seemed to be clinically feasible, safe and able to enhance intratumoral drug delivery in liver tumors resistant to standard chemotherapy.

#### 3.3.2. Sonodynamic Therapy (SDT-Mediated Tumor Cells Death) in Hepatobiliary Cancers

Sonodynamic therapy (SDT) emerges as an innovative LIFU-based modality for liver cancer treatment, offering advantages such as deep tissue penetration, non-ionizing properties, precise controllability, and cost-effectiveness [178,179,180]. SDT employs sonosensitizers to stimulate the production of active reactive oxygen species (ROS), through LIFU, facilitating cancer treatment [181]. ROS can induce ICD and the release of damage-associated molecular patterns (DAMPs). In addition, the maturation of dendritic cells (DCs) results in the transformation of T cells into toxic T cells, thereby achieving tumor killing and long-term immune memory. Furthermore, ROS can reverse the immunosuppressive TME by participating in the maturation of antigen-presenting cells (APCs) and the transformation of anti-inflammatory M2 macrophages into pro-inflammatory M1 macrophages [182,183,184]. The interaction between LIFU and sonosensitizers and the associated acoustic energy transfer mechanism to HCC malignant cells plays a central role in SDT-mediated cell death [165].

The antitumor effect of MBs in combination with sinoporphyrin sodium (DVDMS)-mediated SDT was assessed in vitro and in vivo experiments for HCC treatment using HepG2 cells and Hepa1-6 tumor-bearing mice [166]. While in vitro experiments demonstrated that MBs can enhance SDT-induced ICD, an in vivo experiment that combined SDT with MBs showed a significantly reduced tumor volume, concluding that MBs can markedly improve the anticancer effects of SDT in HCC. Recently, an in vitro and in vivo preclinical study [169] evaluated the therapeutic potential of ICG@C3F8-KL nanobubbles (NBs) combined with SDT for inducing disulfidptosis, a newly identified regulated cell death, which is linked to liver tumor progression. The NBs combined with LIFU effectively induced disulfidptosis and suppressed tumor cell growth without significant toxicity, offering a promising HCC treatment strategy.

#### 3.3.3. SDT-Assisted Immunotherapy (Sono-Immunotherapy) in Hepatobiliary Cancers

Given the treatment resistance and recurrence of HCC due to immunosuppressive TME, researchers focused their work on TME immunomodulation. Based on preclinical studies, SDT demonstrated the ability to generate tumor antigens that can stimulate anti-tumor immune system response [167]. Recently, it was reported that the design of a nanoscale US contrast agent capable of triggering macrophage polarization and ICD for the treatment of HCC through SDT and immunotherapy [185]. The technique known as US-targeted nanobubble destruction (UTND) uses nanosized MBs and LIFU for precise drug delivery. The study successfully developed a macrophage re-educator for SDT-initiated immunotherapy by combining sonosensitizers and Toll-like receptor (TLR) agonists. In in vivo studies, the SDT-initiated immunotherapy using ICG@C3F8-R848 NBs, a sono-immunotherapy mediated controllable composite, demonstrated significant suppression of tumor growth compared to immunotherapy alone. The combination of sonosensitizers, TLR agonists, and SDT has the potential to stimulate anti-tumor immune system response and enhance the HCC treatment efficacy.

In addition, SDT promotes pyroptosis and enhances the immune response to liver cancer [168]. The first report on SDT- induced pyroptosis in liver tumors has designed a phthalocyanine-conjugated mesoporous silicate nanoparticle (PMSN) to augment oxidative stress and induce robust pyroptosis [186]. PMSN-mediated SDT treatment efficiently reduced tumor mass and suppressed residual tumors in treated and distant sites by synergizing with PD-L1 blockade. In addition, loading the chemotherapeutic, doxorubicin, into PMSN intensifies SDT-pyroptotic effects and increases efficacy, suggesting potential for clinical translation.

LIFU can penetrate deeply into tissues and largely target tumor tissue to mediate the cytotoxicity of sonosensitizers. A preclinical study hypothesized that SDT may perform effectively as a cancer vaccine and explored whether SDT can eliminate primary tumors, inhibit metastases, and prevent tumor relapse [167]. Researchers found that HiPorfin (HPD)-induced SDT killed tumor cells, promoted calreticulin expression on the cell surface and elicited immune responses. They also observed that SDT induced functional antitumor vaccination and abscopal effects in H22 tumor-bearing mice, conferring immunological memory and protection against tumor recurrence.

#### 3.3.4. Low-Intensity Focused Ultrasound (LIFU)-Induced Tumor Radiosensitization

Although significant technological advances have been made in radiotherapy, the development of radioresistance in tumor cells remains a challenge, compromising its efficacy [187]. Radiosensitization methods such as chemical radiosensitizers and physical stimuli were developed [188] to overcome tumor radioresistance. LIFU-induced radiosensitization has emerged as a promising method to enhance radiotherapy efficacy as a safer alternative to traditional radiosensitizers and thermotherapies [189], due to its greater tissue penetration depth [190]. It acts by inhibiting DNA repair, diminishing signal transduction, overcoming TME hypoxia, and obstructing angiogenesis [190].

A randomized pilot clinical trial [172] evaluated the safety and efficacy of combining UTMD and transarterial radioembolization (TARE) in 28 HCC patients. Preliminary results showed a greater efficacy prevalence of tumor response, 93% in patients that underwent both UTMD and TARE vs. 50% in the TARE alone group. It was concluded that the combination of UTMD and TARE is feasible and safe and appears to result in improved response of HCC patients to TARE treatment.

There remain some unknowns about the mechanisms driving LIFU-induced radiosensitization that require further investigation. While initial studies have shown promising results, more comprehensive clinical trials are required to confirm its safety and effectiveness in clinical settings.

#### 3.3.5. Quality of Evidence from Studies on LIFU Applications for Hepatobiliary Cancers

**Figure 5:**
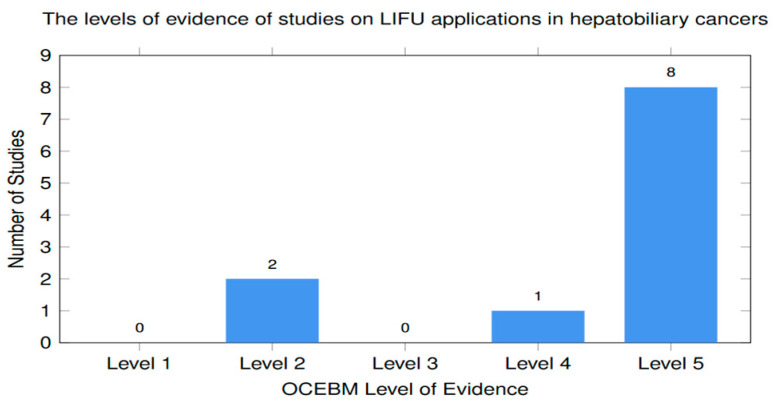
Representation of the levels of evidence of studies on LIFU applications for hepatobiliary cancers.

According to the Oxford Center for Evidence-Based Medicine (OCEBM) hierarchy, the levels of evidence of studies on LIFU applications for hepatobiliary cancers are represented in Figure 5.

While the therapeutic potential of LIFU-mediated applications in hepatobiliary cancers is supported by a growing body of evidence, the current landscape remains heavily weighted toward Level 5 preclinical studies. Despite the innovative mechanisms and significant antitumor effects observed in preclinical models, clinical evidence for LIFU in hepatobiliary oncology is still in its infancy. With only a limited number of Level 2 randomized pilot studies currently available, there is a critical need for high-quality, powered clinical trials. Moving forward, transitioning from ‘proof-of-concept’ to standardized clinical practice will require rigorous investigation into optimal sonication dosing and the long-term immunomodulatory effects of these therapies.

## 4. Concluding Remarks and Future Directions

### 4.1. Concluding Remarks

Soundwave-based therapies have evolved significantly with the advent of modern US-guided technology and MRI imaging, moving from their initial use as thermal ablation to a multifunctional platform for thermal and non-thermal ablation, immunomodulation, and targeted drug delivery. Thus, they may offer a potential multimodal strategy for tumor volume debulking, TME modulation, and potentiation of anticancer drug delivery.

HIFU is a therapeutic opportunity for patients ineligible for surgery and other thermal ablation techniques, while histotripsy, the first non-thermal ablation based on sound waves, adds millimeter precision, safety and its potential for immunostimulatory effects.

HIFU and histotripsy have the potential of noninvasive liver-directed therapies that treat localized, inoperable tumors, either to achieve a complete pathological response (pathological cure) or to reduce the disease to a state where curative surgical resection or transplantation becomes possible.

However, the THERESA and #HOPE4LIVER trials were primarily focused on feasibility, technical success, and safety of histotripsy liver ablation with secondary focuses on short-term efficacy. The high rate of complete tumor ablation serves only as a surrogate marker for long-term prognosis in hepatobiliary malignancies. In the real world, in an off-trial setting, many patients treated with histotripsy had not yet reached 6- or 12-month follow-up. Since no data have been provided on the long-term oncological efficacy of liver tumor ablation by histotripsy [191], the available results should be viewed with caution. In addition, the absence of validated, post-histotripsy imaging criteria introduces a risk of observer bias, and a consensus framework is needed for assessing response to this treatment [192].

According to the IDEAL framework, which allows for a structured assessment of existing data on an innovation, histotripsy is at the transition between IDEAL stages 2a (development) and 2b (exploration). In the development stage, procedures are typically subject to iterative modifications, and studies include a few patients and providers, while in the exploration phase, experience is accumulated in a larger group of patients and providers to establish consensus for future RCT [191].

Preclinical data have suggested a possible abscopal effect of histotripsy due to the lack of thermal denaturation of tumor antigens and systemic antitumor immune priming, but clinical observations are limited to rare case reports. In addition, the abscopal phenomenon has been described since the 1950s with various technologies [191] such as radiotherapy, RFA, MWA, TACE, but it still remains rare and uncertain. The use of histotripsy outside the protocol for immune priming and the pursuit of an abscopal effect are not supported by current evidence [191]. The development of reliable strategies for inducing systemic immune responses through localized FUS-mediated therapies requires a deeper understanding of the mechanisms of the abscopal effect and multidisciplinary collaboration for the optimal sequencing of potentially synergistic combinations with immunomodulators.

Although current data show that histotripsy may be a promising adjuvant for the curative-intent treatment of liver tumors that are not susceptible to established locoregional therapies, its integration into clinical practice requires caution and deliberation [191], depending on the results of ongoing and future clinical trials and the experience accumulated over time.

LIFU-induced targeted drug delivery (sono-chemotherapy), sonodynamic therapy, radiosensitization, immunomodulation of the immunosuppressive TME (sono-immunotherapy), and the potential to enhance the effect of ICIs in these malignancies have shown promising results, mainly in preclinical studies, in improving outcomes and reducing systemic toxicity.

### 4.2. Future Directions

Despite their potential, the translation of FUS-based techniques from research into clinical practice, from technical success and efficacy to improved clinical outcomes, faces several challenges: technical limitations in targeting deep, difficult-to-access tumors, such as biliary tract cancers and some liver segment tumors, high treatment costs, limited availability of these technologies, and the need for large-scale evidence of long-term survival and recurrence data. Acoustically mediated tumor ablations in difficult areas will be enhanced by advances in beam steering and phased-array transducers, allowing for safer targeting near major vessels and access to lesions near the ribs or deep liver segments.

#### Artificial Intelligence (AI)-Based Precise Ablation Targeting, Monitoring HCC Recurrence and Predicting Subclinical Recurrence Risk

Artificial intelligence (AI)-assisted targeting is expected to improve the accuracy of HIFU ablation and histotripsy, reducing off-target lesions. The need for precise intra-procedural monitoring increases when complete tumor ablation involves controlling multiple sonications. To address these challenges, an AI-assisted ultrasound-guided FUS framework was evaluated, incorporating an AI segmentation model with B-mode US imaging. Experimental results showed 93% accuracy in identifying ablated areas, suggesting that AI-assisted US monitoring can significantly improve the accuracy and control of FUS treatments [193]. Although histotripsy achieves submillimeter precision in targeting liver tumors, the efficacy diminishes with increasing tissue depth due to attenuation and aberration of the FUS beam. Advanced phased array guidance systems and AI-assisted aberration correction, using cavitation emission feedback or MRI guidance, have demonstrated up to 80% improvement in focal precision during transcostal liver ablation [156].

In addition, platforms based on bionic organ chips and AI-combined training can simulate tumor-immune microenvironment interactions and rapidly analyze optimal timing associations for targeted immune therapy for liver cancer.

An AI model based on liquid biopsy combined with US elastography will realize HCC recurrence monitoring and predict the risk of subclinical recurrence through the temporal correlation between the ctDNA mutation spectrum and liver stiffness values [194]. AI techniques for the prediction of HCC recurrence after treatment have significant performance. Automatic MRI segmentation can accurately extract tumor volume and radiomics features for early recurrence prediction [194,195]. The shift to precision oncology involves the use of multimodal data models to capture the heterogeneity of cancer development, recurrence, and response to current sequential strategies.

FUS-based cancer treatment modalities have the potential to move from niche applications to a multimodal precision oncology tool. The use of combinations (rather than standalone therapies), such as HIFU with TACE or chemotherapy or histotripsy with immunotherapy, will add value to this promising hybrid strategy.

Since FUS-assisted procedures exhibit dual actions through therapeutic functionality associated with intra- and post-procedural US-imaging guidance, they could have value as a theranostic tool in hepatobiliary interventional oncology.

## 5. Conclusions

FUS-mediated therapies are characterized by distinct profiles of potential benefits, challenges, and limitations. HIFU is mainly applied for ablation of early-stage localized HCC, and tumor reduction surgery for unresectable primary and secondary liver cancer. Despite promising data, the adoption of HIFU in the management of biliary tract cancer remains limited by the lack of large-scale, disease-specific prospective studies. Future research must focus on standardizing ablation protocols and defining the optimal timing of HIFU relative to stent placement and systemic chemotherapy.

Early reported experience with histotripsy ablation of liver tumors supports promising safety and efficacy profiles and its potential as a noninvasive alternative to liver-directed therapy. A current challenge with this new technology is the lack of specific validated post-histotripsy imaging criteria for assessing response to treatment. As histotripsy studies are characterized by heterogeneity, retrospective design, limited cohort size, non-standardized protocols, and short-term follow-up, their results should be interpreted as promising for the treatment of liver tumors.

Early-phase trials and randomized pilot studies have demonstrated the safety and feasibility of LIFU techniques like sono-chemotherapy and ultrasound-targeted microbubble destruction (UTMD)-enhanced radioembolization. However, the evidence base is not yet sufficient to establish definitive clinical protocols for LIFU applications in hepatobiliary malignancies. Future studies should focus on high-level clinical evidence to resolve existing uncertainties regarding treatment reproducibility and the synergistic potential of LIFU with emerging immunotherapies.

Although promising, the available clinical evidence for FUS-mediated therapies in hepatobiliary malignancies consists predominantly of early-stage feasibility studies, retrospective observational cohorts, and non-randomized comparative analyses. Further studies focused on standardized protocols, validation through large-scale, multicenter, prospective randomized clinical trials comparing FUS-based therapies with established treatments, and long-term follow-up of oncological efficacy could define their future role in multimodal oncological strategies.

## Figures and Tables

**Table 1:** Early prospective clinical and feasibility studies of HIFU for liver metastases.

Study	Design	Population	Main Findings	Level of Evidence/Limitations
Yang et al. 2021 [104]	Phase I clinical trial	CLM unsuitable for resection/RFA	Safe, promising tumor response	Early-phase, single-arm, no comparator
Dupré et al. 2015 [105]	Phase I–IIa prospective	Intra-operative CLM cohort	Feasible, accurate ablation	Small selected surgical cohort
Dupré et al. 2023 [106]	Prospective ablate-and-resect	CLM undergoing hepatectomy	23 of 24 CLM were successfully treated (95.8%); safe margins	Surrogate endpoint study
Yang et al. 2024 [107]	Prospective non-randomized	Liver metastases + immunotherapy	Feasible and safe	Non-randomized, exploratory

Abbreviations: CLM = colorectal liver metastases; HIFU = high-intensity focused ultrasound; RFA = radiofrequency ablation.

**Table 2:** Retrospective and non-randomized comparative clinical studies of HIFU for liver metastases.

Study	Design	Population	Main Findings	Key Limitations
Yang et al. 2022 [109]	Multicenter retrospective	238 unresectable CLM	USgHIFU is a feasible and safe alternative treatment	Selection bias; no control arm
Wang et al. 2023 [108]	Comparative clinical study	Small liver metastases	Lower trauma vs. MWA	Non-randomized
Tang et al. 2023 [110]	Propensity-matched retrospective; HIFU+ chemotherapy	Unresectable CLM	Better local control and mPFS with HIFU + systemic therapy	Residual confounding
Wu et al. 2025 [111]	Case–control	Advanced liver cancer (64% metastases)	Improved short-term efficacy with antiangiogenic therapy + HIFU	Mixed population; short follow-up
Wang et al. 2025 [112]	Clinical observational study	CLM	Improved overall survival in the HIFU+ chemotherapy group; lower IL-6	Non-randomized; likely selection bias

Abbreviations: CLM = colorectal liver metastases; HIFU = high-intensity focused ultrasound; MWA = microwave ablation; mPFS = median progression-free survival; USgHIFU = Ultrasound-guided HIFU.

**Table 3:** Potential benefits of High-Intensity Focused Ultrasound (HIFU) compared with Radiofrequency Ablation (RFA) and Microwave Ablation (MWA) in liver cancers.

Domain	HIFU Compared to RFA	HIFU Compared to MWA	References
Invasiveness	Non-invasive, whereas RFA requires electrode insertion into the tumor	Non-invasive vs. percutaneous probe insertion in MWA	Cheung TT et al., 2021 [96], Zhou YF et al., 2011 [118].
Risk of bleeding or infection	Lower risk because the skin and liver capsule are not punctured	Lower procedural bleeding or infection risk than probe-based MWA	Cheung TT et al., 2021 [96], Tsang SH et al., 2021 [119], Zavaglia C et al., 2013 [120]. Ge N et al., 2020 [121].
Tumor seeding risk	HIFU eliminates needle-track tumor seeding reported with RFA (though seeding is already rare in RFA).	HIFU eliminates needle-track tumor seeding risk vs. MWA (though seeding is already rare in MWA).	Cheung TT et al., 2021 [96]
Suitability in poor coagulation or ascites	Can be used in patients unfit for percutaneous ablation (coagulopathy, massive ascites), where RFA is relatively contraindicated	Same benefit vs. MWA	Ng KK et al., 2011 [66], Cheung TT et al., 2012 [56].
Pain and recovery	Shorter time to pain resolution and recovery compared with RFA in comparative studies	HIFU is associated with less post-procedural discomfort than thermal probe techniques	Zhou YF et al., 2011 [118], Sehmbi AS et al., 2021. [61].
Treatment of difficult locations	HIFU is useful for tumors near vessels, bile ducts or subcapsular areas where heat-sink and needle access limit RFA	HIFU avoids antenna placement issues seen with MWA in hard-to-reach sites	Cheung TT et al., 2021 [96], Tsang SH et al., 2021 [119], Wang J et al., 2023 [108].
Repeatability	Repeatable, no upper limit of tissue tolerance to repeated HIFU exposure, no cumulative puncture trauma	Same repeatability benefit vs. MWA	Cheung TT et al., 2021 [96], Kennedy JE et al., 2005 [34].
Efficacy signals (selected studies)	HIFU as a sole strategy has comparable or similar results to RFA	HIFU has benefits compared to MWA for LMs < 3 cm in difficult locations.	Diana M et al., 2016 [60], Wang J et al., 2023 [108].

Abbreviations: HIFU = high-intensity focused ultrasound; RFA = radiofrequency ablation; MWA = microwave ablation; LMs = liver metastases.

**Table 4:** Challenges and Technical Limitations of HIFU for Liver Cancer Treatment.

Challenge	Impact on HIFU Ablation Procedure	Potential Solution
Respiratory Motion	Movement of the liver during breathing can cause targeting errors (off-target damage) and “blur” the focal zone.	Breath-hold techniques or real-time tracking. Use of gated ventilation or breath-hold techniques under general anesthesia to stabilize the target. In conscious patients, real-time MR-tracking and feedback loops allow the beam to follow liver displacement. Aubry et al., 2013 [126]; Gedroyc et al., 2006 [127].
Rib Cage Obstruction	Ribs reflect and absorb ultrasound, risking skin burns and reducing energy delivery to the tumor, [incomplete ablation].	Partial rib resection is reserved for cases where no acoustic window exists, and the tumor is otherwise treatable Alternatively, beam-steering algorithms can “turn off” transducer elements that overlap with ribs to minimize skin heating. Tsang et al., 2021 [119].
Perfusion/Heat Sink	High hepatic blood flow (especially in tumors near large vessels) dissipates heat, preventing the target from reaching necrotic temperatures (>60 °C).	Sequential or combined TACE + HIFU: TACE causes embolization, reducing arterial inflow and the heat-sink effect, which significantly improves the volume of complete necrosis compared to HIFU alone. Hu et al., 2022 [83].
Acoustic Windows	Tumors in the liver dome (Segments 7/8) are often shielded by the lung or diaphragm, making them inaccessible to the FUS beam.	Induction of artificial ascites (saline in the peritoneal cavity) or pleural effusion to displace the lung/diaphragm, creating a safe fluid (transonic pathway) for the FUS beam to reach the dome Rhim et al., 2009 [128], Orsi et al., 2010 [129], Kambadakone et al., 2017 [125].

**Table 5:** Reported safety and efficacy of histotripsy in primary and secondary liver cancers in clinical studies.

Study (First Author)	Study Design/ Population	HCC Cases (Primary Liver Cancer)	Patients/Tumors Treated	Reported Safety and Efficacy/ Outcome
Joan Vidal-Jove (THERESA)—2022. (NCT03741088) [136].	Multicenter phase I feasibility trial; patients with unresectable primary or metastatic liver tumors.	1 HCC (1 of 11 tumors).	8 patients; 11 tumors targeted.	Acute technical success (assessed by MRI at 1 day) achieved in all procedures—11/11 tumors (100%). No device-related adverse events reported through 2 months of follow-up. Median tumor diameter ≈ 1.4 cm.
Mishal Mendiratta-Lala (HOPE4LIVER pooled analysis)—2024. (NCT04572633, NCT04573881) [137].	Prospective, multicenter, single-arm pivotal trials (US + EU/UK) evaluating histotripsy for primary/metastatic liver tumors.	18 participants had HCC (41% of participants).	44 participants pooled (49 tumors enrolled; 44 tumors evaluable for primary technical success).	Technical success: 42/44 tumors (95%; 95% CI 84–100) at ≤36 h. Technique efficacy at 30 days: 30/36 lesions (83%; 95% CI 68–92); maximum tumor diameter 1.5 cm ± 0.6. Procedure-related major complication rate (≤30 days): 3/44 participants (7%). Meets prespecified performance goals.
Cristina Jimenez-Soto (First FDA-approved experience, retrospective)—2025 [138].	Single-center retrospective series of patients treated as early clinical experience after FDA approval.	27 patients treated for primary liver cancers (HCC) and metastases, not specifying the number of HCC cases; downstaging/bridging subgroup includes primary tumors.	9 patients received histotripsy with downstaging or bridging intent, 18 cases as part of a palliative treatment.	Among patients treated with downstaging/bridging intent: 7/8 evaluable (87.5%) showed no lesion growth at ≤3 months. In palliative cohort, growth remained controlled in 3/9 (33%) at ≤3 months. 3 patients successfully downstaged to resection and 5 to transplant.
Ziemlewicz Timothy J. 2025 [139].	The 1-year clinical outcomes of patients enrolled in the #HOPE4LIVER trial.	47 patients treated (19 primary and 28 metastatic liver cancers).	17/19 HCC and 27/28 LMs had multifocal tumors; 52 tumors treated.	Local control rates at 1 year consistent with current locoregional therapies; favorable safety profile; overall survival at 1 year was 73.3% for HCC and 48.6% for LMs, comparable with other liver-directed therapies.
Wehrle, Chase. J. 2025 [140]	The First International Experience with Histotripsy: A Safety Analysis of 230 Cases	230 patients from 9 centers	31 HCC, 199 LMs,	High safety, with only 5.2% (12/230) with only 5.2% (12/230) of patients experiencing complications, 75% of which were minor.

Abbreviations: HCC = hepatocellular carcinoma; LMs = liver metastasis.

**Table 6:** Low-Intensity Focused Ultrasound (LIFU) applications have been reported to be effective in hepatobiliary cancers in preclinical studies.

Application Type	Mechanism of Action	Efficacy/Key Findings	Reference
LIFU Targeted Drug Delivery (Sono-chemotherapy)	LIFU-triggered release of chemotherapy (e.g., Adriamycin) from microbubble-nanoparticle complexes.	Achieved the highest Volume Inhibition Rate (VIR) and longest survival (71 days) in rabbit VX2 liver tumor models.	Gong Y et al., 2016 [162]
LIFU Targeted Drug Delivery (Sono-chemotherapy)	LIFU sonoporation + Albumin-doxorubicin nanoparticle conjugated microbubble (ADMB) to enhance the therapeutic efficiency of chemotherapy.	Doxorubicin loading efficiency was 82.7%; tumor growth was reduced fivefold compared to the control group.	Lee JH et al., 2019 [163]
Low-intensity pulsed ultrasound (LIPUS) Drug Delivery (Sono-chemotherapy)	LIPUS-assisted chemotherapy in cholangiocarcinoma (CCA).	Significantly enhanced gemcitabine and cisplatin penetration; fivefold increase in apoptotic cell death.	Hong S et al. 2025 [164].
SDT + Microbubbles (MBs) Sensitization	Antitumor effect of MBs in combination with sinoporphyrin sodium (DVDMS)-mediated SDT in HCC treatment.	In vitro, MBs enhanced SDT-induced immunogenic cell death; in vivo, SDT + MBs significantly reduced tumor volume.	Yang H et al. 2024 [165].
SDT + Nanobubbles (NBs) Sensitization	SDT + ICG@C3F8-KL nanobubbles for inducing disulfidptosis linked to liver tumor progression.	Effectively induced disulfidptosis (novel cell death mechanism) and suppressed HCC cell growth.	Chen Y et al. 2025 [166].
Sonodynamic Therapy (SDT) (Sono-immunotherapy)	Nanoscale US contrast agent triggered macrophage polarization and immunogenic cell death (ICD) for HCC treatment.	SDT-initiated immunotherapy combining sonosensitizers and TLR agonists; significant tumor suppression.	Chen Y et al., 2023 [167].
Sonodynamic Therapy (SDT) (Sono-immunotherapy)	SDT + PMSN induces pyroptosis in liver tumors, combined with PD-L1 blockade.	PMSN-mediated SDT efficiently reduced tumor mass by synergizing with PD-L1 blockade.	Zhang N et al. 2024 [168]
Sonodynamic Therapy (SDT) (Sono-immunotherapy)	SDT acting as a cancer vaccine; explored if it eliminates tumors and prevents recurrence.	HiPorfin-induced SDT killed tumor cells, promoted calreticulin expression, and elicited abscopal effects.	Zhang Q et al. 2018 [169].

Abbreviations: CCA = cholangiocarcinoma; HCC = hepatocellular carcinoma; LIFU = Low-intensity focused ultrasound; LIPUS = Low-intensity pulsed ultrasound; ICD = immunogenic cell death; MBs = microbubbles.

**Table 7:** Low-Intensity Focused Ultrasound (LIFU) applications have been reported to be effective in hepatobiliary cancers in clinical studies.

Application Type	Mechanism of Action	Efficacy/Key Findings	Reference
LIFU Targeted Drug Delivery (Sono-chemotherapy)	Randomized clinical study: LIFU + MBs + chemotherapy vs. chemotherapy alone in colorectal liver metastasis (CLM).	Safe and feasible; tendency toward larger-volume reduction in LIFU + MBs + chemotherapy group.	Haram M et al. 2023 [170].
Mild hyperthermia induced by LIFU (Phase I TARDOX) (NCT02181075).	Lyso-thermosensitive liposomal doxorubicin (LTLD) triggered by LIFU hyperthermia in unresectable primary and secondary liver tumors.	Feasible and safe; enhanced intra-tumoral drug delivery in tumors resistant to standard chemotherapy.	Lyon PC et al. 2018 [171].
LIFU-induced Radiosensitization	Randomized pilot clinical trial to evaluate the safety/efficacy of UTMD + TARE in HCC patients.	Feasible and safe; tumor response prevalence was 93% in the UTMD + TARE group vs. 50% in TARE alone.	Eisenbrey JR et al. 2021 [172].

Abbreviations: CLM = colorectal liver metastasis; HCC = hepatocellular carcinoma; LIFU = Low-intensity focused ultrasound; MBs = microbubbles; TARE = transarterial radioembolization; UTMD = US-triggered MB destruction.

## Data Availability

No new data were created or analyzed in this study.

## Abbreviations

HCC, hepatocellular carcinoma; CCA, cholangiocarcinoma; CLM, colorectal liver metastases; LMs, liver metastases; TME, tumor microenvironment; FUS, focused ultrasound; US, ultrasound; HIFU, high-intensity focused ultrasound; LIFU, low-intensity focused ultrasound; MRgHIFU, Magnetic Resonance-guided High-Intensity Focused Ultrasound; USgHIFU, Ultrasound-guided High-Intensity Focused Ultrasound; ICD, immunogenic cell death immune checkpoint inhibitors (ICIs); m RECIST, modified Response Evaluation Criteria in Solid Tumors; MWA, microwave ablation; RFA, radiofrequency ablation; PEI, Percutaneous ethanol injection; TACE, transarterial chemoembolization; RCTs, randomized controlled trials; MBO, malignant biliary obstruction; MBs, microbubbles; NBs, nanobubbles; OS, overall survival; ROS, reactive oxygen species; SDT, sonodynamic therapy; UMTD, ultrasound microbubble targeted destruction, DAMPs, damage-associated molecular patterns.
